# Effect and comparison of bone marrow aspirate concentrate on cartilage regeneration and clinical outcomes following high tibial osteotomy and microfracture

**DOI:** 10.1186/s13018-026-06996-w

**Published:** 2026-05-28

**Authors:** Onur Cetin, Abdulkadir Durdi, Tahsin Beyzadeoglu

**Affiliations:** 1 Department of Orthopedics and Traumatology, Istanbul Medipol University, Istanbul, Turkey; 2 Department of Orthopedics and Traumatology, Sancaktepe Education and Training Hospital, Istanbul, Turkey; 3 Department of Orthopedics and Traumatology, Beyzadeoglu Clinic, Istanbul, Turkey

**Keywords:** High tibial osteotomy, Bone marrow aspirate concentrate, Microfracture, Cartilage regeneration

## Abstract

**Objective:**

Medial open-wedge high tibial osteotomy (MOWHTO) is an established joint-preserving procedure for medial compartment knee osteoarthritis associated with varus malalignment. However, while MOWHTO effectively corrects mechanical alignment, it does not directly address biochemical environment of the knee joint or concomitant full-thickness cartilage defects. Biological augmentation strategies such as microfracture (MFX) and bone marrow aspirate concentrate (BMAC) have been proposed to enhance cartilage regeneration and improve clinical outcomes. The purpose of this study was to compare clinical, radiological, and second-look arthroscopic outcomes of patients treated with MOWHTO combined with MFX, with or without BMAC augmentation.

**Methods:**

A retrospective comparative study was conducted on 113 patients with medial compartment knee osteoarthritis who underwent MOWHTO and MFX between 2015 and 2022. All patients were followed-up for a minimum of 2 years. Patients were allocated into two groups: MOWHTO + MFX (Group I, *n* = 52) and MOWHTO + MFX + BMAC (Group II, *n* = 61). Clinical outcomes were evaluated using International Knee Documentation Committee (IKDC) and Lysholm scores. Cartilage regeneration was assessed during second-look arthroscopy at the time of plate removal using the International Cartilage Regeneration Society Cartilage Repair Assessment (ICRS-CRA) and Koshino staging systems. Radiological parameters including femorotibial angle and posterior tibial slope were also analyzed.

**Results:**

Both groups demonstrated significant improvements in clinical and radiological outcomes at final follow-up. However, Group II showed significantly superior cartilage regeneration, with higher mean ICRS-CRA scores compared to Group I (9.50 ± 1.52 vs. 8.62 ± 1.51; *p* = 0.001), as well as ICRS-CRA grades and Koshino stages (*P* = 0.01 and 0.049, respectively). Clinical improvements were also greater in the BMAC group, with significantly higher postoperative IKDC and Lysholm scores and greater score improvements from baseline (all *p* < 0.01). Radiological alignment correction was comparable between groups, with no loss of correction.

**Conclusion:**

While MOWHTO combined with MFX provides effective mechanical realignment and clinical improvement, the addition of BMAC improves cartilage regeneration quality and leads to superior functional outcomes. However, the absolute differences in clinical scores remained below the reported minimal clinically important difference (MCID) thresholds in the literature, suggesting that the clinical relevance of these improvements may be limited. Therefore, while BMAC augmentation appears to enhance structural cartilage regeneration, its routine use should be carefully considered.

**Level of evidence III:**

This journal requires that authors assign a level of evidence to each article. For a full description of these Evidence-Based Medicine ratings, please refer to the Table of Contents or the online Instructions to Authors www.springer.com/00266.

## Introduction

Osteoarthritis (OA) of the knee joint is a degenerative and progressive disease with alterations in the mechanical and biochemical environment leading to cartilage destruction [1–3]. It mostly emerges with varus malalignment and medial compartment arthritis because of excessive contact pressure on the medial side of the knee [4, 5]. It is a common cause of pain and functional limitation in middle-aged and socially active patient population. Even though total joint arthroplasty is a well-known and successful surgery for patients with intractable symptoms and advanced stages of OA, many joint preserving methods are suitable for the treatment of patients with mild symptoms such as PRP injections, mesenchymal stem cell injections of, autologous matrix-induced chondrogenesis, microfracture (MFX), osteotomies around the knee [6–8]. Furthermore, recent studies suggest that combination of surgical interventions such as simultaneous autologous chondrocyte implantation with high tibial osteotomy have favorable outcomes [9].

Medial Opening Wedge High Tibial Osteotomy (MOWHTO) is a well-established joint-preserving procedure by correcting mechanical axis alignment, offloading the degenerated compartment and slope correction [10, 11]. MOWHTO decelerates the progression of OA in medial compartment by redistributing the mechanical stress towards the lateral compartment [12]. Even though ideal mechanical axis can be provided with MOWHTO, altered biochemical environment leading to degradation of cartilage does not ameliorate, and concomitant full thickness cartilage defects remain as they were before and have an undesired impact on the long-term success of the surgery [13, 14]. Therefore, many authors have suggested that biological healing procedures should be considered to augment the cartilage healing process in addition to realignment procedure with the aim of improved long-term outcomes [15–19]. Microfracture (MFX) is commonly used to stimulate cartilage repair by promoting marrow-derived cell infiltration into the defect site. In addition, bone marrow aspirate concentrate (BMAC) is a concentrated source of autologous mesenchymal stem cell (MSC), cytokines and growth factors that may enhance the regenerative potential of cartilage and may improve joint microenvironment [20–22]. Recent studies have demonstrated that intra-articular BMAC injection and MFX techniques combined with MOWHTO is a promising treatment method especially for genu varum deformity with concomitant cartilage defects [23–25].

This study aimed to compare the clinical, radiological and second-look arthroscopic outcomes of patients treated with MOWHTO and MFX with and without BMAC augmentation. We hypothesized that adding BMAC to MOWHTO and MFX may improve the quality of cartilage repair and possibly clinical outcomes at short-term follow-up compared to MOWHTO and MFX alone.

## Materials and methods

### Study design and setting

Ethical approval was obtained from the institutional review board of Istanbul Medipol University (File date and number: 25/06/2025; E-10840098-202.3.02-3963) and conducted in accordance with the Declaration of Helsinki. The participants provided their written informed consent to participate in this study. A total of 171 patients undergoing MOWHTO for medial compartment knee osteoarthritis between 2015 and 2022 were retrospectively screened for this study.

### Participants

Inclusion criteria were as follows;

1. BMI < 35 kg/m2.
2. Varus deformity < 15 degree.
3. No history of any other knee surgery (except partial meniscectomy or repair) or any other severe knee pathology (concomitant ligament pathologies resulting clinical instability, lateral compartment arthritis, contracture of the knee joint, history of infection of the knee joint, rheumatoid arthritis).
4. Patients having their implant removed and had second-look arthroscopic surgery after minimum 1 year from the initial surgery.
5. Minimum 2-year follow-up.
6. All demographic, PROM (Lysholm and International Knee Documentation Committee-IKDC) and radiological data (X-rays and preoperative MRI) available for retrospective screening.

Patients who had bilateral simultaneous knee surgery, loss to follow-up, BMI over 35, K-L grade 4 medial compartment arthrosis (kissing lesions or diffuse full-thickness cartilage loss involving both opposing articular surfaces of the medial compartment), concomitant meniscus root tears and patients who did not consent to a second surgery for implant removal and/or second-look arthroscopy were excluded from the study. In addition, patients with a K-L grade ≥ 2 in the lateral compartment were excluded to avoid the inclusion of patients with advanced lateral compartment osteoarthritis.

All patients were informed that they will be suggested to undergo plate removal after osteotomy union due to potential implant-related irritation and to reduce the risk of hardware-related symptoms in active patients and second-look arthroscopy will be performed simultaneously to evaluate medial chondral pathologies and possible additional procedures.

### Patient selection 

**Fig. 1:**
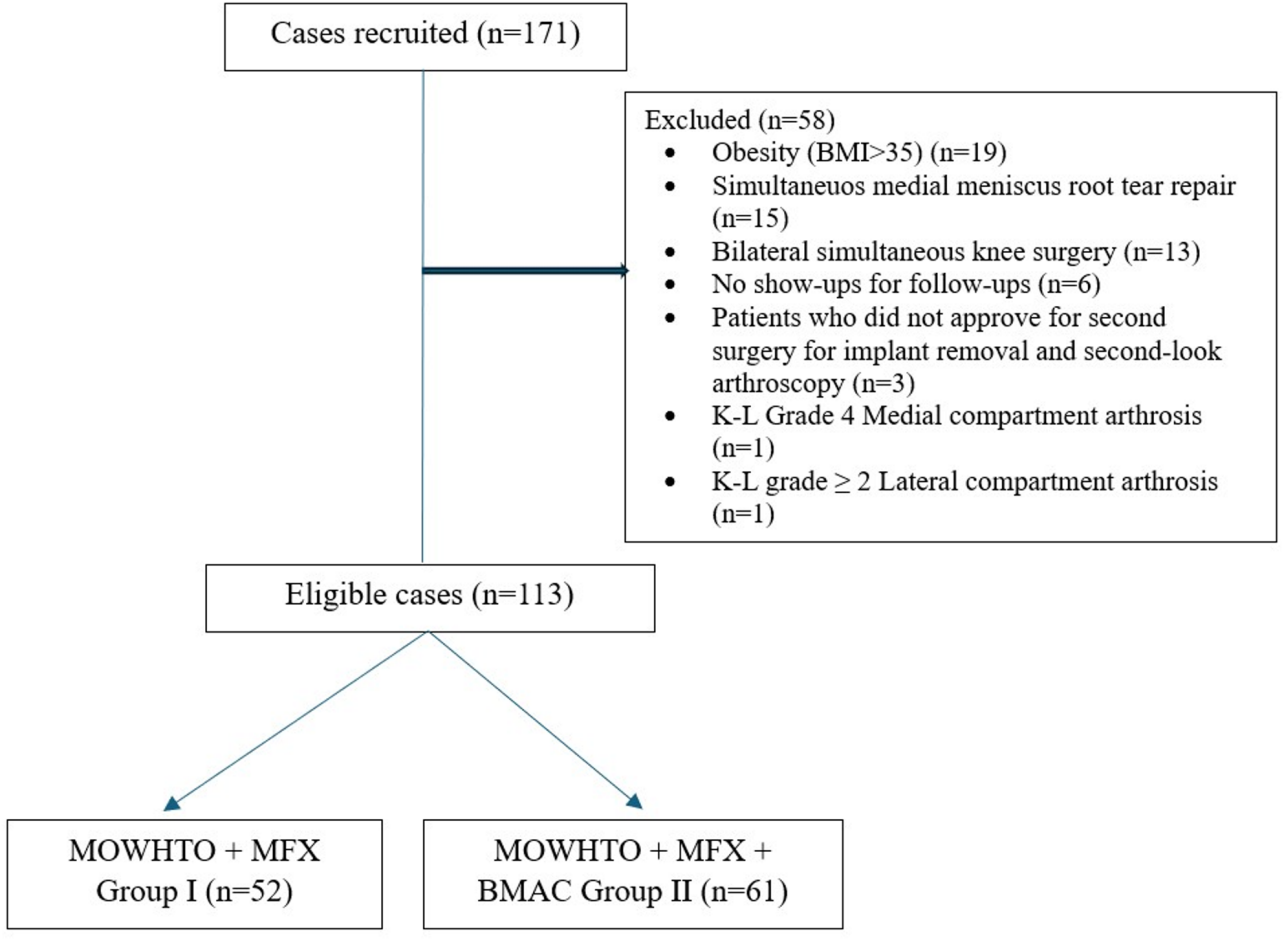
Flowchart of the cohort

A comparative cohort design with baseline comparability was employed. Following application of inclusion and exclusion criteria, patients were allocated into two groups based on the surgical treatment received (MOWHTO + MFX vs. MOWHTO + MFX + BMAC) (Fig. 1).

To minimize selection bias, baseline demographic and clinical variables including age, sex, body mass index (BMI), Kellgren–Lawrence grade, cartilage lesion size, and follow-up duration were compared between groups and demonstrated no statistically significant differences (all *p* > 0.05) (Table 1).

**Table 1:** Demographic and clinical characteristics of the cohort (Bold value indicates statistical significance)

	Group I	Group II	Mean difference (95% CI)	P
Age (years)	52.31 ± 6.44	51.82 ± 6.07	0.49 (−2.83 to 1.86)	0.681
Sex (Female) (no. [%])	29 (55.8%)	35 (57.4%)	–	0.864
BMI (kg/m^2^)	27.42 ± 3.27	26.98 ± 3.11	-0.44 (-1.63 to 0.76)	0.468
Side (Right) (no. [%])	27 (51.9%)	30 (49.2%)	—	0.775
Follow-up Duration (Months)	27.31 ± 3.48	27.89 ± 3.77	−0.58 (−0.77 to 1.92)	0.397
Interval between index and second-look surgery (Months)	12.46 ± 0.61	12.85 ± 0.72	−0.39 (−0.75 to -0.03)	**0.034**
Defect Size of MFC (cm^2^)	5.21 ± 1.03	5.34 ± 1.07	−0.13 (−0.52 to 0.25)	0.485
K-L Grade of Medial Compartment (II/III)	32/20	39/22	–	0.991

Additionally, multivariable regression analysis was performed to adjust for potential confounders and to assess the independent effect of BMAC augmentation on clinical outcomes.

### Description of treatment and surgery

**Fig. 2:**
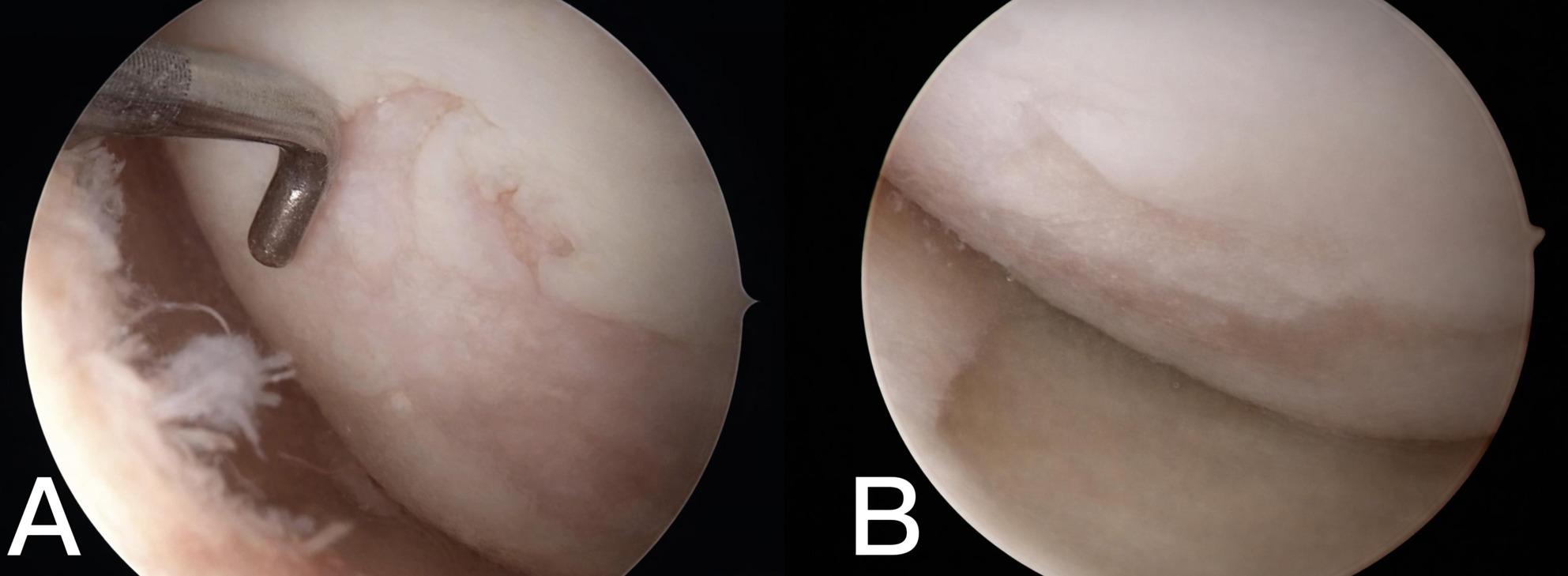
**A**; Initial evaluation of the cartilage with the help of probe.** B**; Second-look arthroscopic evaluation of the regenerated cartilage

All procedures were performed by a single senior surgeon. In both groups, standard arthroscopic surgery was performed. All patients evaluated for their cartilage defects and defect sizes were noted by fellow surgeon to overcome potential bias (Fig. 2A). After debridement of defective cartilage, MFX was performed in a standard fashion. If there were unstable meniscal tear fragments, debridement was performed and if needed, circumferential meniscus repair was performed.

After arthroscopic surgery was completed, a medial uniplanar open wedge high tibial osteotomy was performed. Neutral alignment of the mechanical axis was aimed for each individual. Knee alignment was checked intraoperatively using fluoroscopy to evaluate the femoro-tibial angle. No grafts were used in the osteotomy line of any patient. A medial locking plate system was used after completion of osteotomy.

In addition to these procedures, in Group II, approximately 120 ml of bone marrow was aspirated using the Bio-MAC Bone Marrow Aspiration Catheter system^®^ (Biologic Therapies, Ocala, FL, USA) from the ipsilateral iliac spine. After centrifugation of bone marrow aspirate through the Bio-SPIN™ Magellan^®^ centrifuge (Biologic Therapies Ocala, FL, USA), the concentrated bone marrow was collected which was approximately 6–8 ml. It was then applied to the defect site right after application of MFX under dry arthroscopic view.

Plate removal and second-look arthroscopy were performed for all patients at minimum 1-year after the initial surgery. To reduce observer bias, arthroscopic recordings were independently evaluated for ICRS-CRA grading and Koshino staging by a fellow surgeon who was not involved in the surgical procedures and who was blinded to the treatment group (Fig. 2B). Right after the arthroscopy, plate removal was performed.

### Aftercare

Rehabilitation was standardized for both groups, and all individuals had the same rehabilitation protocol. Rehabilitation program was conducted at the same center with a team of experienced physiotherapists three times a week for eight weeks. Passive and active range-of-motion (ROM) exercises started on the first postoperative day as tolerated. Patients mobilized with two Canadian crutches in partial weight bearing manner for the first 4 weeks. Full weight-bearing was allowed approximately 6 to 8 weeks postoperatively. Home exercises were assigned to strengthen the knee, hip, and ankle using isometric and isotonic exercises, as well as balance proprioception exercises.

After plate removal and second-look arthroscopy, full-weight bearing with Canadian crutches was allowed on the 1st postoperative day. ROM and isometric exercises were started right after the surgery and isotonic exercises were started at the first postoperative week.

### Primary outcome measures

Cartilage defect size evaluated macroscopically at first arthroscopic surgery. Cartilage regeneration and quality of the regenerated cartilage was assessed according to the ICRS-CRA (Table 2) and Koshino staging system (Table 3) at second-look arthroscopy at the time of plate removal.

**Table 2:** ICRS CRA grading system

	Points
Degree of defect repair	
Level with surrounding cartilage	4
75% repair of defect depth	3
50% repair of defect depth	2
25% repair of defect depth	1
0% repair of defect depth	0
Integration to border zone	
Complete integration with surrounding cartilage	4
Demarcating border < 1mm	3
Three-fourths of graft integrated with surrounding cartilage, one-fourth with a notable border > 1mm	2
One-half of graft integrated with surrounding cartilage, one-half with a notable border > 1mm	1
From no contact to one-fourth of graft integrated with surrounding cartilage	0
Macroscopic appearance	
Intact, smooth surface	4
Fibrillated surface	3
Small, scattered fissures or cracks	2
Several small, or few but large fissures	1
Total degeneration of grafted area	0
Overall repair assessment	
Grade I: normal	12
Grade II: nearly normal	8–11
Grade III: abnormal	4–7
Grade IV: severely abnormal	1–3

**Table 3:** Koshino staging system

Stage of regeneration	
Stage A	No regeneration or no repair
Stage B	Pink fibrous tissue with or without partial coverage with white fibrocartilage
Stage C	1. Total coverage with white overgrown cartilage 2. Total coverage with white even smooth cartilage

Clinical assessments were recorded, and functional improvements were evaluated using Patient Reported Outcome Measures (PROMs) and Range of Motion (ROM). PROMs were assessed at initial examination and at final follow-up using International Knee Documentation Committee (IKDC) and Lysholm Knee Scoring Scale.

### Secondary outcome measures

Orthoroentgenogram, weight bearing 2-plane knee X-ray and Rosenberg X-ray were obtained for all patients. Femorotibial Angle (FTA) and posterior tibial slope (PTS) were measured for all patients preoperatively and at the final follow up after plate removal. Medial compartment arthritis was evaluated on weight bearing knee X-rays and Rosenberg X-ray using Kellgren-Lawrence (K-L) grade system. In addition, MRI was evaluated prior to surgery for evaluating soft tissue and cartilage defects.

### Statistical analysis

All statistical analyses were performed using SPSS 24 (IBM SPSS Corp.; Armonk, NY, US). Continuous variables were first assessed for normality using visual distribution inspection and were compared between the two treatment groups using Welch’s t-test, given the possibility of unequal variances. Results are presented as mean ± standard deviation (SD), together with absolute mean differences and corresponding 95% confidence intervals (CI). Categorical variables, including sex distribution, operated side, and Kellgren–Lawrence grades, were analyzed using chi-square tests or Fisher’s exact test when expected cell counts were < 5.

ICRS CRA scores were analyzed as continuous variables using Welch’s t-test, whereas categorical ICRS CRA grades and Koshino stages were compared using chi-square tests. PROM improvement (Δ) was calculated as postoperative minus preoperative scores and compared using Welch’s* t*-test.

To account for potential confounding factors, multivariable linear regression analyses were performed to evaluate the independent association between BMAC augmentation and clinical outcomes. Separate regression models were constructed for postoperative Lysholm score, postoperative IKDC score, Lysholm improvement, and IKDC improvement. Covariates entered into the models included age, sex, BMI, Kellgren–Lawrence grade, cartilage lesion size, and time to second-look arthroscopy or follow-up duration as appropriate. For postoperative outcome models, the corresponding preoperative PROM score was also included as a covariate to control for baseline differences.

Regression coefficients (β) with 95% confidence intervals (CI) were calculated. Statistical significance was defined as *p* < 0.05.

## Results

Of the 171 initially screened patients, 58 were excluded based on predefined criteria (Fig. 1). The main reasons for exclusion were BMI over 35 kg/m^2^ (*n* = 19), simultaneous medial meniscus root tear repair (*n* = 15), bilateral simultaneous knee surgery (*n* = 13), loss to follow-up (*n* = 6), lack of second-look arthroscopy or implant removal (*n* = 3), Kellgren–Lawrence grade 4 (*n* = 1), and lateral compartment arthrosis (K–L ≥ grade 2) (*n* = 1). After applying these criteria, 113 patients were included in the final analysis.

There were no statistically significant differences between the groups regarding age, sex, BMI, lesion size of the medial femoral condyle, Kellgren–Lawrence grade, or follow-up duration (all *p* > 0.05), although the interval between the index procedure and second-look arthroscopy was slightly longer in Group II (mean difference 0.39 months, 95% CI -0.75 to -0.03; *p* = 0.034) (Table 1).

Intra-observer analysis demonstrated that both groups showed significant improvement in articular cartilage regeneration at final follow-up compared to initial examination. However, inter-observer analysis of second-look arthroscopic assessment demonstrated significantly superior cartilage quality in the BMAC group. The mean ICRS CRA score was higher in Group II compared with Group I (9.50 ± 1.52 vs. 8.62 ± 1.51; mean difference 0.88, 95% CI 0.39 to 1.38; *p* = 0.001) (Table 4). In categorical analysis, the distribution of ICRS CRA grades and Koshino staging differed significantly between groups in favor of the BMAC group (*p* = 0.010, *p* = 0.049, respectively) (Table 4).

**Table 4:** Second-look arthroscopic evaluation of cartilage regeneration (Bold values indicate statistical significance)

	Group I	Group II	P
**ICRS CRA Score**	8.62 ± 1.51	9.50 ± 1.52	**0.001**
**ICRS CRA Categorical Distribution**			**0.010**
Grade 1	6 (11.5%)	14 (23.0%)	
Grade 2	21 (40.4%)	27 (44.3%)	
Grade 3	17 (32.7%)	15 (24.6%)	
Grade 4	8 (15.4%)	5 (8.1%)	
**Koshino Staging System**			**0.049**
Stage A	23 (44.2%)	18 (29.5%)	
Stage B	22 (42.3%)	28 (45.9%)	
Stage C	7 (13.5%)	15 (24.6%)	

Radiological outcomes confirmed effective correction of mechanical alignment in both groups without any loss of correction. Intra- and inter-group analysis of pre- and postoperative FTA and PTS, as well as their changes, were similar between groups, with no statistically significant differences (all *p* > 0.05) (Table 5).

**Table 5:** Clinical and radiological results and postoperative complication rates (Bold values indicate statistical significance)

		Group I Mean ± SD	Group II Mean ± SD	Mean difference (95% CI)	P
Femorotibial Angle (FTA) (Valgus)	Pre-op (°)	6.83 ± 1.72	6.74 ± 1.69	0.09 (−0.56 to 0.74)	0.784
	Final Follow Up (°)	2.32 ± 1.21	2.28 ± 1.18	0.04 (−0.39 to 0.47)	0.854
	ΔFTA (°)	4.51 ± 1.46	4.46 ± 1.43	0.05 (−0.50 to 0.60)	0.856
Posterior Tibial Slope (PTS)	Pre-op (°)	8.41 ± 2.11	8.36 ± 2.05	0.05 (−0.72 to 0.82)	0.894
	Final Follow Up (°)	9.27 ± 2.1	9.34 ± 2.22	−0.07 (−0.89 to 0.75)	0.866
	ΔSlope (°)	0.86 ± 1.01	0.98 ± 1.08	−0.12 (−0.53 to 0.29)	0.562
		Group I Mean ± SD	Group II Mean ± SD	Mean Difference (95% CI)	P
IKDC	Preoperative	36.39 ± 2.79	35.14 ± 2.10	1.25 (0.32 to 2.18)	**0.009**
	Postoperative Final Follow Up	73.02 ± 5.36	76.34 ± 5.08	−3.33 (−5.29 to −1.37)	**0.001**
	ΔIKDC	36.63 ± 3.92	41.20 ± 4.05	−4.58 (−6.10 to −3.06)	** < 0.001**
Lysholm	Preoperative	60.86 ± 5.37	57.96 ± 4.67	2.90 (1.00 to 4.80)	**0.003**
	Postoperative Final Follow Up	81.23 ± 4.90	84.13 ± 5.02	−2.90 (−4.73 to −1.07)	**0.002**
	ΔLysholm	20.37 ± 4.37	26.16 ± 5.11	−5.80 (−7.71 to −3.89)	** < 0.001**
		Group I N (%)	Group II N (%)	P	
Complications	Major	4 (7.7%)	4 (6.5%)	1.00	
	Minor	3 (5.8%)	9 (14.7%)	0.13	

Patient-reported outcomes improved markedly in both cohorts; however, clinical gains were more pronounced in the BMAC group. Final Lysholm scores were higher in Group II (84.13 ± 5.02 vs. 81.23 ± 4.90; *p* = 0.002), with a greater increase from baseline (Change 26.16 ± 5.11 vs. 20.37 ± 4.37; *p* < 0.001). Similarly, final IKDC scores were significantly higher in the BMAC group (Final IKDC: 73.02 ± 5.36 vs. 76.34 ± 5.08; *p* = 0.001; change: 36.63 ± 3.92 vs. 41.20 ± 4.05; *p* < 0.001) (Table 5).

### Multivariable regression analysis

To determine whether the observed differences in clinical outcomes were independent of baseline characteristics, multivariable linear regression analyses were performed adjusting for potential confounders including age, sex, BMI, Kellgren–Lawrence grade, cartilage defect size, and time to second-look arthroscopy or follow-up duration as appropriate. After adjustment, BMAC augmentation remained independently associated with improved postoperative clinical outcomes. In the adjusted models, BMAC use was associated with a 4.06-point increase in postoperative Lysholm score (95% CI 2.29 to 5.83; *p* < 0.001) and a 5.42-point increase in postoperative IKDC score (95% CI 3.94 to 6.90; *p* < 0.001). Similarly, BMAC augmentation was independently associated with greater improvements in PROM scores, with a 5.33-point increase in Lysholm improvement (95% CI 3.34 to 7.32; *p* < 0.001) and a 4.97-point increase in IKDC improvement (95% CI 3.45 to 6.48; *p* < 0.001). These findings indicate that the association between BMAC augmentation and improved functional outcomes persisted after controlling for potential confounding variables.

### Complications

Postoperative complications were grouped as minor and major complications. Major complications were hinge fracture. There was no deep surgical infection in any of the patients. Minor complications were swelling of the knee, pain in the BMAC harvesting area, superficial infection or hematoma of the incision area (Table 5). 8 patients, 4 in each group had Takeuchi type 1 lateral hinge fracture and treated conservatively. In each group, 1 patient had superficial surgical site infection with no culture positivity without any clinically significant change. Minor complications which was swelling of the knee, temporary pain around the knee or pain at the BMAC harvest area were seen in 3 patients in Group I and 9 patients in Group II.

## Discussion

One of the main findings of this study is that BMAC augmentation provides superior cartilage regeneration following MOWHTO and MFX, as demonstrated by second-look arthroscopy. However, another important finding was that although the BMAC-augmented group showed statistically significant improvements in clinical scores, these differences remained below the minimum clinically important difference (MCID) thresholds. Therefore, while BMAC augmentation after MOWHTO and MFX resulted in visibly better cartilage regeneration, this structural improvement did not translate into a clinically meaningful advantage.

Previous studies have reported the minimum clinically important difference (MCID) for IKDC scores to be approximately 9.8–11.5 points and for Lysholm scores approximately 4.2–12.5 points [26–30]. In the present study, the mean between-group differences were below or near these thresholds, suggesting that although BMAC augmentation demonstrated statistical superiority, the clinical magnitude of improvement may be modest.

Although the present study did not include direct biochemical or cytokine measurements, the observed improvements in cartilage morphology in the BMAC group can be interpreted in the context of the known biological activity of BMAC which may enhance chondrogenesis, extracellular matrix synthesis, and proteoglycan deposition. Therefore, the superior macroscopic cartilage appearance observed during second-look arthroscopy in the BMAC group likely reflects these underlying biochemical processes, even though they were not directly quantified in this study.

Knee OA is characterized by wear of chondral surfaces as a result of biochemical and mechanical changes [1, 2]. It is a time-dependent pathology which mostly starts with medial compartment pain. Varus malalignment progresses to an excessive pressure over medial compartment and mostly resulting in meniscal degeneration tear and cartilage wear [4, 5].

However, mechanical changes are not the sole pathophysiology of arthritis. Many patients suffer from arthritis without mechanical changes. Microenvironment of the joint may change in time, and biochemical pathologies arise such as overexpression of catabolic cytokines, synovial inflammation, decreased extracellular matrix components and those changes may cause chondrocyte apoptosis and result in arthritis [31, 32]. Hence, both mechanical and biochemical pathologies of knee joint should be considered if knee preservation is planned, especially in middle-aged population with mild-severe OA symptoms.

Several studies suggest MOWHTO as one of the primary surgical option especially in middle-aged patients dealing with genu varum, associated medial compartment arthritis and difficulty in active sports participation [5, 33–37]. This procedure ensures improved mechanical alignment in patients with constitutional varus malalignment by performing precise preoperative planning for osteotomy [38]. By redistributing load line by shifting excessive pressure over medial compartment to lateral compartment, progression of OA, cartilage wear and meniscal degeneration may be postponed in short to mid-term period [5, 35, 36, 39]. Even though MOWHTO improves mechanical axis of the knee joint, it may not primarily provide a better biochemical joint environment. Some studies demonstrated that cartilage regeneration is insufficient in MOWHTO and to have a better long-term outcome, augmentation for cartilage regeneration should be considered [34, 40, 41].

Microfracture is generally recommended for relatively small cartilage defects when lesion size is less than 2.5–4 cm^2^. In larger defects, other cartilage procedures should be considered if concomitant knee pathology cannot be easily addressed [42, 43]. In the present study, the main pathology which was varus deformity was corrected with MOWHTO and MFX was performed for concomitant chondral pathologies even though area of the defect was over 4 cm^2^. The main decision behind the MFX was if the main pathology which was varus deformity was corrected, less invasive surgery may be enough for the cartilage defect.

In addition, orthobiologics such as BMAC are considered a viable option especially for cartilage regeneration [44, 45]. Orthobiologics have ability to differentiate to chondrocytes and have immune-modulatory and anti-inflammatory effects with the help of its contents such as anabolic cytokines, anti-catabolic cytokines and other types of interleukins [1, 46].

A study by He-jin et al. demonstrated that HTO + MFX was clinically sufficient and BMAC combination has no superiority clinically over a follow-up period of 5 years. However, they also concluded that BMAC application resulted in better cartilage regeneration [24]. Furthermore, Kim et al. demonstrated that mesenchymal stem cell (MSC) augmentation over HTO + MFX has favorable clinical outcomes over solely HTO + MFX treatment group [47].

Gobbi et al. compared MFX with BMAC hyaluronic acid scaffolding for 5-year follow-up. During follow-up period, both groups demonstrated significant improvement in 2-year period. However, MFX group normal or near-normal rate of PROMs decreased from 64 to 28%. In contrast, in BMAC-HA group, PROMs maintained improved state for up to 5 year follow-up [23].

Saw et al. studied cartilage regeneration histologically for 8 patients underwent HTO and subsequent injection of peripheral blood stem cell injections for 5 weeks. In addition to clinical improved for all patients, chondral biopsy which was obtained in second-look arthroscopy demonstrated that cartilage resembling native cartilage was observed with a significant amount of proteoglycan and type II collagen [48].

In this study, we demonstrated that simultaneous BMAC application after mechanical correction with MOWHTO + MFX may contribute to a favorable cartilage regeneration. Arthroscopically near normal chondral surface can be obtained and it may speculate that superior clinical outcomes can be obtained with a longer period of survivorship. However, improved clinical scores demonstrated in the BMAC group remained below the MCID thresholds. Therefore, structural improvement demonstrated in the cartilage in the BMAC augmented group did not translate into better clinical scores.

For another perspective, various cartilage procedures such as mosaicplasty, autologous chondrocyte implantation etc. can be considered after MOWHTO. However, these procedures have inconsistent results with increased complication rates, especially in relatively elderly patients [18, 34, 49, 50]. Furthermore, untreated accompanying cartilage pathologies tend to more rapidly progress to arthritis even though MOWHTO was performed and may ended up with a need for total knee arthroplasty, which has lower functional results compared to primary knee arthroplasty [51]. Considering less invasive procedures for cartilage pathologies when performing MOWHTO, many studies reported simultaneous BMAC augmentation for cartilage regeneration has almost none or minor complications and improves functional results [23, 24, 50].

In this study, it has been demonstrated that none of the patients in the BMAC group has no major complication associated with the BMAC augmentation. Complications associated with BMAC application were limited to temporary swelling of the knee and temporary mild to moderate pain in the harvesting area.

### Limitations

There are some limitations for this study which should be addressed. Firstly, retrospective design is a major limitation of our study. Secondly, the ICRS and Koshino grading at second-look arthroscopy were assessed by one fellow surgeon and Koshino grading p-value of 0.049 lacks robustness. Third, a longer period of follow-up was more preferable to demonstrate BMAC application whether changes the longer period of survivorship of the knee joint or not. Finally, cartilage regeneration was solely assessed using second-look arthroscopy. Advanced imaging modalities such as MRI-based cartilage scoring systems (e.g., MOCART) may have provided additional objective evaluation of cartilage repair tissue. Additionally, although baseline characteristics between groups were comparable and multivariable regression analyses were performed to adjust for potential confounders, formal propensity score matching was not performed. Therefore, residual selection bias related to treatment allocation cannot be completely excluded.

## Conclusion

In conclusion, both MOWHTO combined with MFX and MOWHTO combined with MFX and BMAC resulted in substantial improvements in clinical and radiological outcomes. BMAC augmentation was associated with superior cartilage regeneration and statistically better PROM scores. However, the absolute differences in IKDC and Lysholm scores remained below the reported minimal clinically important difference (MCID) thresholds in the literature, suggesting that the clinical relevance of these improvements may be limited. Therefore, while BMAC augmentation appears to enhance structural cartilage regeneration, its routine use should be carefully considered against the additional cost and invasiveness of the procedure, particularly when the expected short-term functional gains are modest. Careful patient selection may be more beneficial in specific subgroups considering cost-effectiveness and invasiveness.

## Data Availability

The datasets used and/or analysed during the current study are available from the corresponding author on reasonable request (Dr Tahsin Beyzadeoglu, tbeyzade@superonline.com).

## References

[1] Iijima H, Isho T, Kuroki H, Takahashi M, Aoyama T. Effectiveness of mesenchymal stem cells for treating patients with knee osteoarthritis: a meta-analysis toward the establishment of effective regenerative rehabilitation. npj Regenerative Med. 2018;3:15. 10.1038/s41536-018-0041-8 PMC6141619 30245848

[2] Tan SHS, Kwan YT, Neo WJ, et al. Outcomes of High Tibial Osteotomy With Versus Without Mesenchymal Stem Cell Augmentation: A Systematic Review and Meta-analysis. Orthop J Sports Med. 2021;9:23259671211014840. 34212066 10.1177/23259671211014840 PMC8216369

[3] Hunter DJ, Bierma-Zeinstra S. Osteoarthr Lancet. 2019;393:1745–59. 10.1016/S0140-6736(19)30417-9 31034380

[4] Fujisawa Y, Masuhara K, Shiomi S. The effect of high tibial osteotomy on osteoarthritis of the knee. An arthroscopic study of 54 knee joints. Orthop Clin North Am. 1979;10:585–608. 460834

[5] Tanamas S, Hanna FS, Cicuttini FM, Wluka AE, Berry P, Urquhart DM. Does knee malalignment increase the risk of development and progression of knee osteoarthritis? A systematic review. Arthritis Care Research: Official J Am Coll Rheumatol. 2009;61:459–67. 10.1002/art.24336 19333985

[6] Redler LH, Caldwell J-M, Schulz BM, Levine WN. Management of articular cartilage defects of the knee. Physician Sportsmed. 2012;40:20–35. 10.3810/psm.2012.02.1948 22508248

[7] Siebold R, Karidakis G, Feil S, Fernandez F. Second-look assessment after all-arthroscopic autologous chondrocyte implantation with spheroides at the knee joint. Volume 24. Arthroscopy: Knee Surgery, Sports Traumatology; 2016. pp. 1678–85. 10.1007/s00167-015-3822-2 26704798

[8] Pintore A, Notarfrancesco D, Zara A, et al. Intra-articular injection of bone marrow aspirate concentrate (BMAC) or adipose-derived stem cells (ADSCs) for knee osteoarthritis: a prospective comparative clinical trial. J Orthop Surg Res. 2023;18:350. 37170296 10.1186/s13018-023-03841-2 PMC10176826

[9] Franceschi F, Longo UG, Ruzzini L, Marinozzi A, Maffulli N, Denaro V. Simultaneous arthroscopic implantation of autologous chondrocytes and high tibial osteotomy for tibial chondral defects in the varus knee. Knee. 2008;15:309–13. 18541430 10.1016/j.knee.2008.04.007

[10] Loia MC, Vanni S, Rosso F, et al. High tibial osteotomy in varus knees: indications and limits. Joints. 2016;4:098–110. 10.11138/jts/2016.4.2.098 PMC4993553 27602350

[11] Martinez de Albornoz P, Leyes M, Forriol F, Del Buono A, Maffulli N. Opening wedge high tibial osteotomy: plate position and biomechanics of the medial tibial plateau. Knee Surg Sports Traumatol Arthrosc. 2014;22:2641–7. 23624677 10.1007/s00167-013-2517-9

[12] Spahn G, Hofmann GO, von Engelhardt LV, Li M, Neubauer H, Klinger HM. The impact of a high tibial valgus osteotomy and unicondylar medial arthroplasty on the treatment for knee osteoarthritis: a meta-analysis. Knee surgery, sports traumatology. Arthroscopy. 2013;21:96–112. 10.1007/s00167-011-1751-2 22076053

[13] Sprenger TR, Doerzbacher JF. Tibial osteotomy for the treatment of varus gonarthrosis: survival and failure analysis to twenty-two years. JBJS. 2003;85:469–74. 12637433

[14] Tang WC, Henderson IJ. High tibial osteotomy: long term survival analysis and patients’ perspective. Knee. 2005;12:410–3. 16046133 10.1016/j.knee.2005.03.006

[15] Ferruzzi A, Buda R, Cavallo M, Timoncini A, Natali S, Giannini S. Cartilage repair procedures associated with high tibial osteotomy in varus knees: clinical results at 11 years’ follow-up. Knee. 2014;21:445–50. 24507767 10.1016/j.knee.2013.11.013

[16] Cavallo M, Sayyed-Hosseinian S-H, Parma A, Buda R, Mosca M, Giannini S. Combination of high tibial osteotomy and autologous bone marrow derived cell implantation in early osteoarthritis of knee: a preliminary study. Archives Bone Joint Surg. 2018;6:112. PMC5867354 29600263

[17] Harris JD, McNeilan R, Siston RA, Flanigan DC. Survival and clinical outcome of isolated high tibial osteotomy and combined biological knee reconstruction. Knee. 2013;20:154–61. 23477914 10.1016/j.knee.2012.12.012

[18] Pascale W, Luraghi S, Perico L, Pascale V. Do microfractures improve high tibial osteotomy outcome? Orthopedics. 2011;34:e251–5. 21717984 10.3928/01477447-20110526-06

[19] Sterett WI, Steadman JR, Huang MJ, Matheny LM, Briggs KK. Chondral resurfacing and high tibial osteotomy in the varus knee: survivorship analysis. Am J Sports Med. 2010;38:1420–4. 20375366 10.1177/0363546509360403

[20] Oreffo RO, Cooper C, Mason C, Clements M. Mesenchymal stem cells: lineage, plasticity, and skeletal therapeutic potential. Stem Cell Rev. 2005;1:169–78. 17142852 10.1385/SCR:1:2:169

[21] Barry F, Murphy M. Mesenchymal stem cells in joint disease and repair. Nat Rev Rheumatol. 2013;9:584–94. 23881068 10.1038/nrrheum.2013.109

[22] Andia I, Maffulli N. Mesenchymal stromal cell products for intra-articular knee injections for conservative management of osteoarthritis. Ther Adv Musculoskelet Dis. 2021;13:1759720x21996953. 33680097 10.1177/1759720X21996953 PMC7897835

[23] Gobbi A, Whyte GP. One-stage cartilage repair using a hyaluronic acid–based scaffold with activated bone marrow–derived mesenchymal stem cells compared with microfracture: five-year follow-up. Am J Sports Med. 2016;44:2846–54. 27474386 10.1177/0363546516656179

[24] Jin Q-H, Chung Y-W, Na S-M, Ahn H-W, Jung D-M, Seon J-K. Bone marrow aspirate concentration provided better results in cartilage regeneration to microfracture in knee of osteoarthritic patients. Volume 29. Arthroscopy: Knee Surgery, Sports Traumatology; 2021. pp. 1090–7. 10.1007/s00167-020-06099-x 32556433

[25] Meena A, D’Ambrosi R, Farinelli L, et al. Should I add orthobiologics to my knee osteotomy practice? A systematic review. J ISAKOS. 2024;9:100282. 38851324 10.1016/j.jisako.2024.06.001

[26] Qiao Y, Wu C, Wu X, et al. The Value of Minimal Clinically Important Difference, Substantial Clinical Benefit, and Patient-Acceptable Symptomatic State for Commonly Used Patient-Reported Outcomes in Recurrent Patellar Instability Patients After Medial Patellofemoral Ligament Reconstruction and Tibial Tubercle Transfer. Arthroscopy: J Arthroscopic Relat Surg. 2024;40:115–23. 10.1016/j.arthro.2023.06.042 37419222

[27] Ogura T, Ackermann J, Mestriner AB, Merkely G, Gomoll AH. The Minimal Clinically Important Difference and Substantial Clinical Benefit in the Patient-Reported Outcome Measures of Patients Undergoing Osteochondral Allograft Transplantation in the Knee. Cartilage. 2021;12:42–50. 30463426 10.1177/1947603518812552 PMC7755969

[28] Maheshwer B, Wong SE, Polce EM, et al. Establishing the Minimal Clinically Important Difference and Patient-Acceptable Symptomatic State After Arthroscopic Meniscal Repair and Associated Variables for Achievement. Arthroscopy: J Arthroscopic Relat Surg. 2021;37:3479–86. 10.1016/j.arthro.2021.04.058 33964390

[29] Retzky JS, Shah AK, Neijna AG, Rizy ME, Gomoll AH, Strickland SM. Defining the minimal clinically important difference for IKDC and KOOS scores for patients undergoing tibial tubercle osteotomy for patellofemoral pain or instability. J Exp Orthop. 2024;11:e12115. 39076849 10.1002/jeo2.12115 PMC11284960

[30] Gomoll A, Merkely G, Barbieri Mestriner A, Ackermann J, Ogura T. Minimal Clinically Important Differences and Substantial Clinical Benefit in Patient-Reported Outcome Measures after Autologous Chondrocyte Implantation. Cartilage. 2020;11:412–22. 30221977 10.1177/1947603518799839 PMC7488950

[31] Lories RJ, Luyten FP. The bone–cartilage unit in osteoarthritis. Nat Rev Rheumatol. 2011;7:43–9. 21135881 10.1038/nrrheum.2010.197

[32] Bijlsma JW, Berenbaum F, Lafeber FP. Osteoarthritis: an update with relevance for clinical practice. Lancet. 2011;377:2115–26. 21684382 10.1016/S0140-6736(11)60243-2

[33] Kim J-H, Kim H-J, Lee D-H. Survival of opening versus closing wedge high tibial osteotomy: a meta-analysis. Sci Rep. 2017;7:7296. 28779084 10.1038/s41598-017-07856-8 PMC5544741

[34] Kim K-I, Seo M-C, Song S-J, Bae D-K, Kim D-H, Lee SH. Change of chondral lesions and predictive factors after medial open-wedge high tibial osteotomy with a locked plate system. Am J Sports Med. 2017;45:1615–21. 28291955 10.1177/0363546517694864

[35] Bode G, von Heyden J, Pestka J, et al. Prospective 5-year survival rate data following open-wedge valgus high tibial osteotomy. Volume 23. Sports Traumatology, Arthroscopy: Knee Surgery; 2015. pp. 1949–55. 10.1007/s00167-013-2762-y 24241123

[36] Salzmann GM, Ahrens P, Naal FD, et al. Sporting activity after high tibial osteotomy for the treatment of medial compartment knee osteoarthritis. Am J Sports Med. 2009;37:312–8. 19022990 10.1177/0363546508325666

[37] Gougoulias N, Khanna A, Maffulli N. Sports activities after lower limb osteotomy. Br Med Bull. 2009;91:111–21. 19549634 10.1093/bmb/ldp023

[38] Mederake M, Eleftherakis G, Schüll D, et al. The gap height in open wedge high tibial osteotomy is not affected by the starting point of the osteotomy. BMC Musculoskelet Disord. 2023;24:373. 37170106 10.1186/s12891-023-06478-8 PMC10173475

[39] Hernigou P, Medevielle D, Debeyre J, Goutallier D. Proximal tibial osteotomy for osteoarthritis with varus deformity. A ten to thirteen-year follow-up study. J bone joint Surg Am volume. 1987;69:332–54. 3818700

[40] Schuster P, Geßlein M, Schlumberger M, et al. Ten-year results of medial open-wedge high tibial osteotomy and chondral resurfacing in severe medial osteoarthritis and varus malalignment. Am J Sports Med. 2018;46:1362–70. 29589953 10.1177/0363546518758016

[41] Jung W-H, Takeuchi R, Chun C-W, et al. Second-look arthroscopic assessment of cartilage regeneration after medial opening-wedge high tibial osteotomy. Arthroscopy: J Arthroscopic Relat Surg. 2014;30:72–9. 10.1016/j.arthro.2013.10.008 24384273

[42] Gopinatth V, Jackson GR, Touhey DC, et al. Microfracture for medium size to large knee chondral defects has limited long-term efficacy: A systematic review. J Exp Orthop. 2024;11:e70060. 39429888 10.1002/jeo2.70060 PMC11490187

[43] Camp CL, Stuart MJ, Krych AJ. Current concepts of articular cartilage restoration techniques in the knee. Sports Health. 2014;6:265–73. 24790697 10.1177/1941738113508917 PMC4000472

[44] Migliorini F, Pilone M, Simeone F, Jeyaraman M, Bell A, Maffulli N. Progress in the clinical use of bone marrow aspirate concentrate for knee osteoarthritis: an expert opinion. J Orthop Surg Res. 2025;20:1065. 41372742 10.1186/s13018-025-06509-1 PMC12690796

[45] Migliorini F, Pilone M, Ascani J, Schäfer L, Jeyaraman M, Maffulli N. Management of knee osteoarthritis using bone marrow aspirate concentrate: a systematic review. Br Med Bull. 2025;153:ldae016. 39506910 10.1093/bmb/ldae016

[46] Freitag J, Bates D, Wickham J, et al. Adipose-derived mesenchymal stem cell therapy in the treatment of knee osteoarthritis: a randomized controlled trial. Regen Med. 2019;14:213–30. 30762487 10.2217/rme-2018-0161

[47] Kim YS, Koh YG. Comparative matched-pair analysis of open-wedge high tibial osteotomy with versus without an injection of adipose-derived mesenchymal stem cells for varus knee osteoarthritis: clinical and second-look arthroscopic results. Am J Sports Med. 2018;46:2669–77. 30080423 10.1177/0363546518785973

[48] Saw K-Y, Anz A, Jee CS-Y, Ng RC-S, Mohtarrudin N, Ragavanaidu K. High tibial osteotomy in combination with chondrogenesis after stem cell therapy: a histologic report of 8 cases. Arthroscopy: J Arthroscopic Relat Surg. 2015;31:1909–20. 10.1016/j.arthro.2015.03.038 26008951

[49] Bode G, Ogon P, Pestka J, et al. Clinical outcome and return to work following single-stage combined autologous chondrocyte implantation and high tibial osteotomy. Int Orthop. 2015;39:689–96. 25300396 10.1007/s00264-014-2547-z

[50] Kumagai K, Akamatsu Y, Kobayashi H, Kusayama Y, Saito T. Mosaic osteochondral autograft transplantation versus bone marrow stimulation technique as a concomitant procedure with opening-wedge high tibial osteotomy for spontaneous osteonecrosis of the medial femoral condyle. Arthroscopy: J Arthroscopic Relat Surg. 2018;34:233–40. 10.1016/j.arthro.2017.08.244 29102568

[51] Cerciello S, Vasso M, Maffulli N, Neyret P, Corona K, Panni AS. Total knee arthroplasty after high tibial osteotomy. Orthopedics. 2014;37:191–8. 24762146 10.3928/01477447-20140225-08

